# A UDP-X Diphosphatase from *Streptococcus pneumoniae* Hydrolyzes Precursors of Peptidoglycan Biosynthesis

**DOI:** 10.1371/journal.pone.0064241

**Published:** 2013-05-15

**Authors:** Krisna C. Duong-Ly, Hyun Nyun Woo, Christopher A. Dunn, WenLian Xu, Andrej Babič, Maurice J. Bessman, L. Mario Amzel, Sandra B. Gabelli

**Affiliations:** 1 Department of Biophysics and Biophysical Chemistry, Johns Hopkins University School of Medicine, Baltimore, Maryland, United States of America; 2 Department of Biology and McCollum-Pratt Institute, Johns Hopkins University, Baltimore, Maryland, United States of America; 3 School of Pharmaceutical Sciences, University of Geneva, University of Lausanne, Geneva, Switzerland; 4 Department of Medicine, Johns Hopkins University School of Medicine, Baltimore, Maryland, United States of America; Oak Ridge National Laboratory, United States of America

## Abstract

The gene for a Nudix enzyme (*SP_1669*) was found to code for a UDP-X diphosphatase. The *SP_1669* gene is localized among genes encoding proteins that participate in cell division in *Streptococcus pneumoniae.* One of these genes, *MurF*, encodes an enzyme that catalyzes the last step of the Mur pathway of peptidoglycan biosynthesis. Mur pathway substrates are all derived from UDP-glucosamine and all are potential Nudix substrates. We showed that UDP-X diphosphatase can hydrolyze the Mur pathway substrates UDP-N-acetylmuramic acid and UDP-N-acetylmuramoyl-L-alanine. The 1.39 Å resolution crystal structure of this enzyme shows that it folds as an asymmetric homodimer with two distinct active sites, each containing elements of the conserved Nudix box sequence. In addition to its Nudix catalytic activity, the enzyme has a 3′5′ RNA exonuclease activity. We propose that the structural asymmetry in UDP-X diphosphatase facilitates the recognition of these two distinct classes of substrates, Nudix substrates and RNA. UDP-X diphosphatase is a prototype of a new family of Nudix enzymes with unique structural characteristics: two monomers, each consisting of an N-terminal helix bundle domain and a C-terminal Nudix domain, form an asymmetric dimer with two distinct active sites. These enzymes function to hydrolyze bacterial cell wall precursors and degrade RNA.

## Introduction

Peptidoglycan is an essential component of cell walls that allows bacteria to maintain shape and structural integrity under conditions of osmotic stress. The peptidoglycan is constantly remodeled, recycled, degraded, and synthesized during cell growth and cell division. These properties render the peptidoglycan layer a crucial player in the pathogenicity of many prokaryotes [1].

Peptidoglycan biosynthesis begins with the Mur pathway, which takes place in the cytoplasm [2]. The Mur pathway has been an area of interest because its enzymes provide targets for antibiotic design. One of these enzymes, MurF, catalyzes the last step of peptidoglycan synthesis, linking D-Ala-D-Ala to UDP-N-acetylmuramoyl-L-Ala-D-Glu-L-Lys. Since substrates of peptidoglycan biosynthesis, and more specifically the Mur pathway, are UDP derivatives, they are potential substrates of Nudix hydrolases, enzymes that hydrolyze the phosphoanhydride bond of Nucleoside diphosphates linked to some other moiety, x [3]. The only conserved sequence shared among members of the Nudix superfamily is the “Nudix box consensus sequence” GX_5_EX_7_REUXEEXGU (also called “Nudix motif” or “Nudix box”) where X is any amino acid and U is a hydrophobic amino acid, typically isoleucine, leucine, or valine [3]. Nudix enzymes that hydrolyze simple nucleotides such as ATP and dCTP have been characterized but more complex compounds such as UDP-N-acetylmuramic acid and the other Mur pathway substrates are also potential substrates [2]. Nudix enzymes are present in viruses, prokaryotes, and eukaryotes of various levels of metabolic complexity [3], [4].

The *Streptococcus pneumoniae* gene *SP_1669* encodes a Nudix hydrolase. The gene is located in an operon containing *MurF*, suggesting that it plays a role in the Mur pathway of peptidoglycan biosynthesis by hydrolyzing Mur pathway substrates. This enzyme contains a modified Nudix signature sequence, GX_5_YX_7_KEUXEEXGU, in which a conserved glutamate/arginine pair that forms a hydrogen bond in the canonical motif is substituted by a tyrosine/lysine pair (position 7 and 13 of the signature sequence). To determine whether the enzyme encoded by *SP_1669* can actually hydrolyze Mur pathway substrates, we measured the activity of the enzyme against UDP-N-acetylmuramic acid and UDP-N-acetylmuramoyl-L-alanine, two intermediates in the Mur pathway. The enzyme robustly hydrolyzes these compounds, indicating that it is a UDP-X diphosphatase (UDP-Xase) that hydrolyzes substrates of the form of UDP linked to some moiety X (UDP-X). The 1.39 Å resolution structure of this enzyme shows that it is an asymmetric homodimer with two distinct catalytic sites, one “open” and one “closed-in”.

Based on its similarity with the CDP-Chase from *Bacillus cereus,* a Nudix enzyme that hydrolyzes CDP-choline and has, in addition, an RNA exonuclease activity [5], we tested UDP-Xase against RNA substrates and showed that it indeed has a 3′5′ exonuclease activity. The UDP-Xase from *Streptococcus pneumoniae*, may be a member of a novel Nudix hydrolase family whose members fold as asymmetric homodimers and act on bacterial cell wall precursors as well as RNA.

## Materials and Methods

### Wild-type Enzyme Expression and Purification

The gene encoding the *S. pneumoniae* TIGR4 UDP-Xase *SP_1669* was amplified from genomic DNA using PCR. NdeI and BamHI sites were placed at the start and end of the gene. The amplified DNA was cloned into a TOPO vector (Invitrogen), purified, cut with NdeI and BamHI restriction enzymes and ligated into the pET-24a (Novagen) vector. The resultant plasmid was used to transform BL21(DE3) (Novagen).

Cells were grown at 37°C in LB media. When an OD_600_ of 0.9 was reached, 1 mM IPTG was added to induce expression. After overnight growth at 22°C, the cells were centrifuged, washed in 50 mM Tris pH 7.5, 1 mM EDTA, and 0.1 mM DTT (TED buffer), reharvested, and stored at −80°C.

The over-expressed protein was released upon thawing of the cells. The cell extract was adjusted to 33% ammonium sulfate saturation and allowed to precipitate for 30 minutes at 4°C. Precipitated protein was harvested by centrifugation at 4°C for 30 minutes at 17200×g on a GSA rotor (Sorvall). TED buffer was used to resuspend the precipitated protein. The remaining soluble protein was brought up to 50% ammonium sulfate saturation and the above procedure was repeated to harvest and resuspend precipitated protein. The supernatant after this step was brought up to 30% glycerol (v/v) and loaded onto a Sephadex, G-100 (2.5×50 cm) gel filtration column equilibrated with TED buffer containing 100 mM NaCl. Fractions from the largest A_280_ peak were pooled and centrifuged using a CentriPrep concentrator (Millipore) to a final concentration of 8 mg/mL. SDS-PAGE of the purified protein suggested that it was >95% pure (Figure S1).

### Site-directed Mutagenesis

All mutants were generated using the QuikChange Site-Directed Mutagenesis Kit (Stratagene). Forward and reverse primers for the E113A mutation were 5′-TCCAACAGAAAATATTCTCAAGGCAATTGAAGAAGAAACCGGTTTTA-3′ and 5′-TAAAACCGGTTTCTTCTTCAATTGCCTTGAGAATATTTTCTGTTGGA-3′, respectively. Forward and reverse primers for the E162A mutation were 5′-TGGACAATTCCAAGAAAATCAAGCAATTGCTGACCTTCAATTTTTTG-3′ and 5′-CAAAAAATTGAAGGTCAGCAATTGCTTGATTTTCTTGGAATTGTCCA-3′, respectively. The PCR products were used to transform into GC5 cells (Gene Choice). Plasmid DNA isolated from transformed colonies was sequenced to check for the presence of the desired mutations. Mutant proteins were expressed using the protocol used for the wild-type.

### Purification of UDP-Xase E113A and E162A Mutants

Cells expressing proteins containing mutations were lysed by microfludization. The lysate was brought up to 30% ammonium sulfate saturation, proteins were precipitated, and then resuspended in TED buffer. The remaining lysate was brought to 60% ammonium sulfate saturation and proteins were precipitated and resuspended, brought to 90% ammonium sulfate saturation and precipitated and resuspended again. The proteins of interest were present in the 60% and 90% ammonium sulfate precipitates. These protein fractions were further purified by hydrophobic interaction chromatography using a Phenyl FF 16/10 High Sub column (GE Healthcare) followed by anion exchange chromatography on a Source Q column (GE Healthcare) and size exclusion chromatography on a Sephacryl S200 16/60 column (GE Healthcare). Purity after each step was assessed by SDS-PAGE; proteins were frozen at −80°C until use.

### Nudix Activity Assay

Relative activity assays were performed in 50 µL reaction mixtures containing 50 mM Tris pH 8.4, 5 mM MgCl_2_, 2 mM substrate, 1 unit of calf intestinal alkaline phosphatase (CIP), and 200 nM Nudix enzyme (UDP-Xase). All NDP-X substrates were purchased from Sigma with the exception of UDP-N-acetylmuramic acid and UDP-N-acetylmuramoyl-L-Ala which were synthesized as described previously [6], [7]. The Nudix enzyme converts the CIP-insensitive Nudix substrates to CIP-reactive substrates, which release two molecules of orthophosphate upon reaction with CIP. After incubation for 15 minutes at 37°C, reactions were quenched by the addition of 30 µL of 100 mM EDTA, and inorganic orthophosphate was quantified by the method of Fiske and Subbarow [8] as modified by Ames and Dubin [9].

Kinetic assays were carried out in 230 µL reaction mixtures containing 50 mM Tris pH 8.4, 5 mM MgCl_2_, 2 mM substrate, 1 unit of calf intestinal alkaline phosphatase (CIP), 100 nM Nudix enzyme (UDP-Xase) and 0 to 16 mM substrate (UDP-glucose or UDP-N-acetylmuramic acid or UDP-N-acetylmuramoyl-L-Ala). For each substrate concentration, 50 µL aliquots were taken every 2 minutes for a total of 6 minutes. Each aliquot was immediately quenched as described above and inorganic orthophosphate was quantified as described above. All measurements were done in triplicate and initial rates were fit by non-linear least squares to the Michaelis-Menten equation.

### Protein Crystallization

Crystals of the *S. pneumoniae* UDP-Xase were grown by hanging drop vapor diffusion with a 1 mL reservoir containing 0.1 M Bis-Tris pH 5.5, 0.1–0.3 M Li_2_SO_4_·H_2_O, and 23–26% PEG-3350 (w/v). One µL of reservoir was added to 1 µL of 8 mg/mL protein in TED buffer with 0.10 M NaCl to form the drop. Crystals grew at 20°C in 1–4 days. Prior to data collection, crystals were transferred to reservoir solution supplemented with 10% glycerol (v/v) and flash-frozen in liquid nitrogen.

For phasing, crystals of wild-type UDP-Xase were transferred to a sitting drop containing reservoir solution with 1 mM HgCl_2_. After 2 days of soaking, the crystals showed sufficient derivatization for phasing. Derivatized crystals were frozen prior to X-ray data collection in a manner similar to that used for the native crystals.

### Structure Determination and Refinement

Data for native protein crystals were collected at the Advanced Photon Source (APS) on the LRL-CAT beam line 31A. A single anomalous diffraction (SAD) dataset of a HgCl_2_-derivatized crystal was collected at the National Synchrotron Light Source (NSLS) beamline X6A at the Hg peak (wavelength of 1.0062 Å). Indexing and data reduction were carried out with HKL2000 [10].

The positions of all three anomalously diffracting atoms were determined with the program SOLVE and used for SAD phasing [11]–[15]. After density modification and initial model building at 2.9 Å using the program RESOLVE [16]–[18], some protein strands and helices were still difficult to trace. Non-crystallographic symmetry (NCS) averaging with the DM (density modification) program of the CCP4 suite [19] was used to extend the phases to 2.5 Å. The axis of symmetry was defined using visible elements of the dimer of the C-terminal domains in the initial model. The map generated with the improved phases after NCS averaging [19] allowed manual tracing of the rest of the protein and was followed by iterative cycles of manual model building with the programs O [20], [21] and Coot [22] and refinement with Refmac5 in the CCP4 suite [19]. This model of the HgCl_2_-derivatized protein was used to initiate refinement for the native protein, which diffracted to a higher resolution. Model building was completed with iterative cycles of manual model building with O [20], [21] and refinement with Refmac5 [19]. The final refined model only contained residues in the allowed (95.4%) or the additional favored (4.6%) regions of the Ramachandran plot.

### RNA Exonuclease Assay

Preparation of 5′- and 3′- radiolabeled RNA substrates and assays of RNase activity were carried out as described earlier [5], [23], [24]. To determine the presence of RNase activity and the directionality of this activity, reactions were carried out in 50 mM Tris pH 8.25, 5 mM MgCl_2_, 1 mM DTT, and 0.25 µM UDP-Xase with 40 nM 5′- or 3′-end labeled RNA; water was used in place of enzyme for the negative controls. For the RNase assay involving the mutants, 0.5 µM UDP-Xase was used. For competition assays with unlabeled RNA, 0.25 µM UDP-Xase and 20 nM 5′-end labeled RNA was used and the unlabeled RNA was varied from 0 to 1980 nM. For the competition assays with UDP-glucose, either 0, 5, or 10 mM UDP-glucose was used in the reaction mixture. 10 µL timepoints were taken and quenched with 95% formamide (v/v), 25 mM EDTA, 0.02% bromophenol blue (w/v), and 0.02% cyanol blue (w/v). Products from the reaction were visualized on a urea denaturing gel containing 10% acrylamide (w/v). 2 µL samples were loaded. Gels were dried for 2 hours with heat and vacuum. Gel was exposed overnight with a phosphorimager screen and scanned with the Typhoon imager (GE Healthcare). Gel quantification was performed using ImageQuant (Molecular Dynamics).

### Model of UDP-N-acetylmuramoyl-L-alanine Binding

UDP-N-acetylmuramoyl-L-alanine was docked manually into the “closed-in” active site of the UDP-Xase. The resulting model was optimized by energy minimization restraining residues farther than 4.5 Å from the substrate using the Molecular Operating Environment [25].

### Small Angle X-ray Scattering

Small-angle X-ray scattering (SAXS) data were collected for the E113A and E162A mutants at the National Synchrotron Light Source Beamline X9 with a Mar 165 CCD detector arranged as previously described [26]. Data were processed using the in-house beamline-specific software [26]. Protein concentrations of 4.5 and 6 mg/mL were used. Samples were centrifuged for 10 minutes at 20,000×g on a microcentrifuge. 20 µL of protein was used for each measurement and all scattering data were collected in triplicate. For each sample, the replicates were averaged and a buffer scattering profile was also measured and subtracted from the data.

Data with q≤0.4 Å^−1^ were selected using the program PRIMUS [27]. The value of D_max_ was estimated by optimizing the P(r) pair distributions using the program GNOM. R_g_ was also calculated using GNOM [28]. The goodness of fit (χ^2^) to the wild-type UDP-Xase structure (PDB ID 4HFQ) was calculated using CRYSOL [29].

For each sample, 10 molecular envelopes were generated and averaged using the program DAMAVER [30], [31]. Alignment and superposition of the atomic coordinates to the envelopes were performed using the program SUPCOMB13 [32]. Scattering, and P(r) were drawn using Gnuplot [33].

### Data Deposition

Atomic coordinates for the *S. pneumoniae* UDP-Xase have been deposited in the Protein Data Bank, www.pdb.org, with the accession code 4HFQ.

## Results and Discussion

### Genomic Context of the SP_1669 Gene

**Figure 1:**
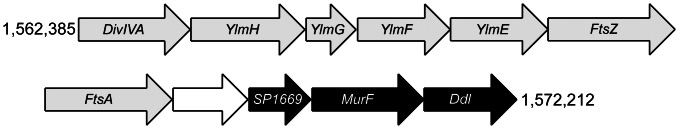
Genomic context of *SP_1669*. *SP_1669* is located in an operon that contains the genes *MurF* and *Ddl* (shaded in black). This operon is located upstream of an operon encoding genes in cell division including *DivIVA*, *YlmH*, *YlmG*, *YlmF*, *YlmE*, *FtsZ*, and *FtsA* (shaded in grey).

*SP_1669*, the gene for a putative Nudix hydrolase in the *S. pneumoniae* genome, was shown using the DOOR database [34] to be located within an operon containing the gene encoding D-alanine-D-alanine synthetase A (*Ddl*), the enzyme that joins two D-alanines to form a dipeptide, and the gene encoding MurF, the enzyme that carries out the addition of a D-alanine-D-alanine dipeptide to UDP-N-acetylmuramoyl-L-alanine-D-glutamate-L-lysine in the peptidoglycan biosynthesis pathway. The genomic localization of the gene *SP_1669* and its Nudix hydrolase sequence, suggest that the protein may hydrolyze, for example, UDP-N-acetylmuramoyl-L-alanine-D-glutamate-L-lysine and other substrates of the Mur pathway. Interestingly, the operon containing *SP_1669* is upstream of another operon containing genes that code for proteins involved in cell division including *DivIVA*, *YlmH*, *YlmG*, *YlmF*, *FtsZ*, and *FtsA* (Figure 1). This is significant because the processes of peptidoglycan biosynthesis and cell division are highly intertwined in bacteria [35].

### Activity and Kinetics of the Protein Encoded by SP_1669 against Mur Pathway Substrates

**Figure 2:**
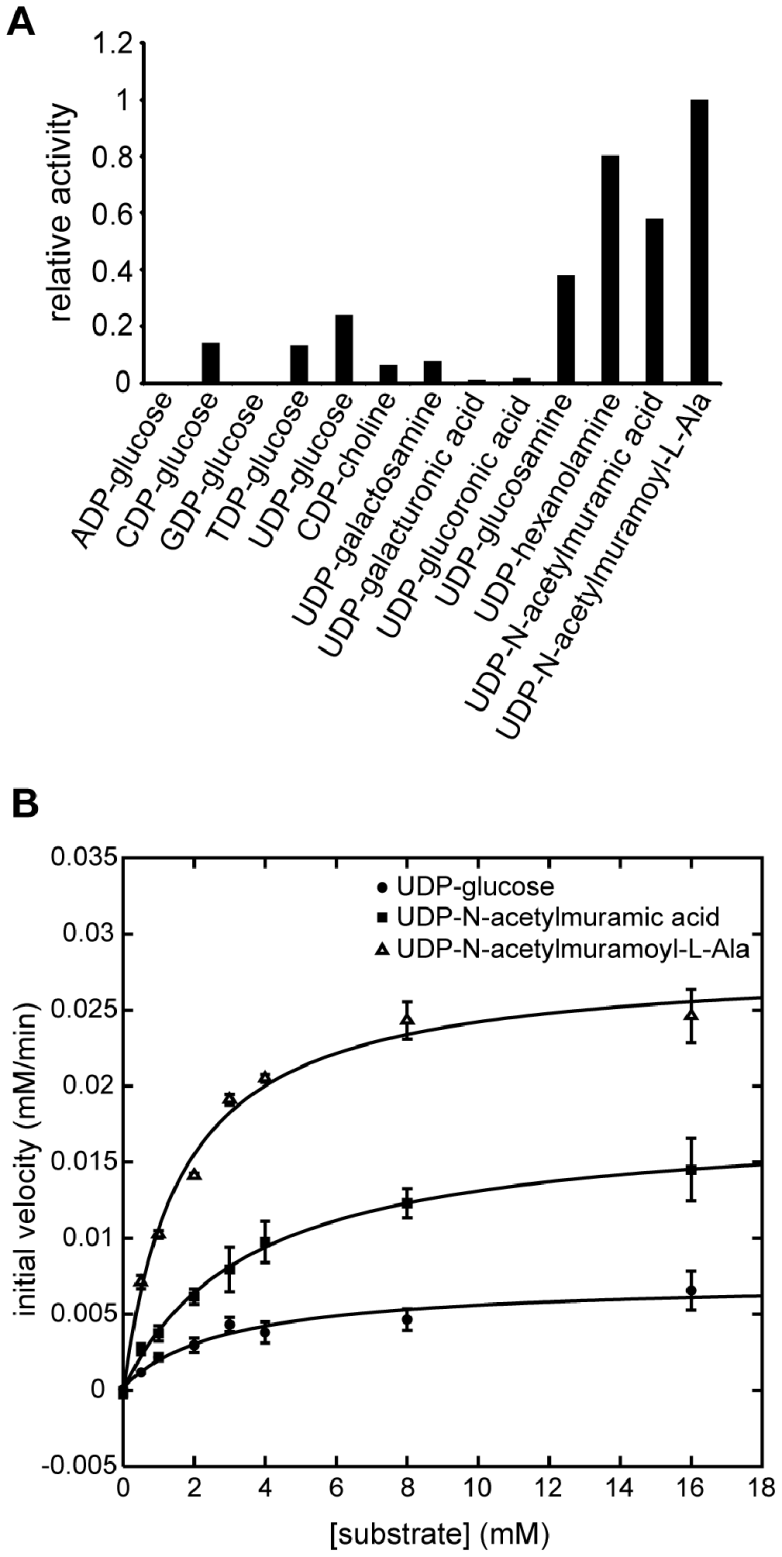
Nudix substrate hydrolysis by UDP-X diphosphatase. (A) Relative hydrolysis of Nudix substrates by UDP-X diphosphatase. The level of hydrolysis of various Nudix substrates is plotted relative to that of UDP-N-acetylmuramoyl-L-Ala. (B) Kinetics of hydrolysis of UDP-glucose, UDP-N-acetylmuramic acid and UDP-N-acetylmuramoyl-L-Ala. Rates were measured in triplicate. Error bars represent standard deviations. Non-linear least-squares fits to the Michaelis-Menten were performed to extract the kinetic parameters in Table 1.

The genomic location of the *SP_1669* gene suggests that its gene product hydrolyzes substrates in the Mur pathway. All of these potential substrates are uridine diphosphates linked to some other moiety X (UDP-X). To test whether or not UDP-X substrates are favored over other nucleoside diphosphates linked to some other moiety X (NDP-X), we recombinantly expressed the *SP_1669* gene product, purified it (Figure S1), and measured the relative hydrolysis of several potential Nudix substrates by this enzyme. We found that UDP-X substrates are preferred over other NDP-X substrates (Figure 2A). We observed the highest amount of hydrolysis for UDP-N-acetylmuramoyl-L-Ala, a Mur pathway substrate (Figure 2A). Since the enzyme encoded by *SP_1669* acts against multiple substrates in the form of UDP-X, we refer to it as a UDP-X diphosphatase (or UDP-Xase).

We also measured the kinetics of hydrolysis by UDP-Xase of two Mur pathway substrates: UDP-N-acetylmuramic acid and UDP-N-acetylmuramoyl-L-Ala. (We were not able to synthesize other Mur pathway substrates.) The kinetics of hydrolysis of UDP-glucose, a known substrate of other Nudix hydrolases [36], [37] was measured as a control. Of the three tested substrates, UDP-N-acetylmuramoyl-L-Ala has the lowest K_M_ (1.6 mM) and the highest k_cat_ (4.7 sec^−1^), suggesting that this is the preferred substrate (Table 1). UDP-N-acetylmuramic acid (k_cat_ 3.0 sec^−1^) is another potential substrate for UDP-Xase (Figure 2B and Table 1). UDP-Xase was also able to hydrolyze UDP-glucose, although it did so at a slightly lower rate than either UDP-N-acetylmuramoyl-L-Ala or UDP-N-acetylmuramic acid.

**Table 1:** Kinetic parameters for hydrolysis of UDP-glucose and Mur pathway substrates.

Substrate	K_M_ (mM)	V_max_ (µM/min)	k_cat_ (s^−1^)	k_cat_/K_M_ [(mol/L) ^−1^s^−1^]
UDP-glucose	2.9±1.1	7.0±0.8	1.2±0.1	400±200
UDP-N-acetylmuramic acid	3.6±0.4	17.7±0.5	3.0±0.1	800±100
UDP-N-acetylmuramoyl-L-Ala	1.6±0.3	28.3±1.3	4.7±0.2	2900±700

### Structure of UDP-Xase

**Figure 3:**
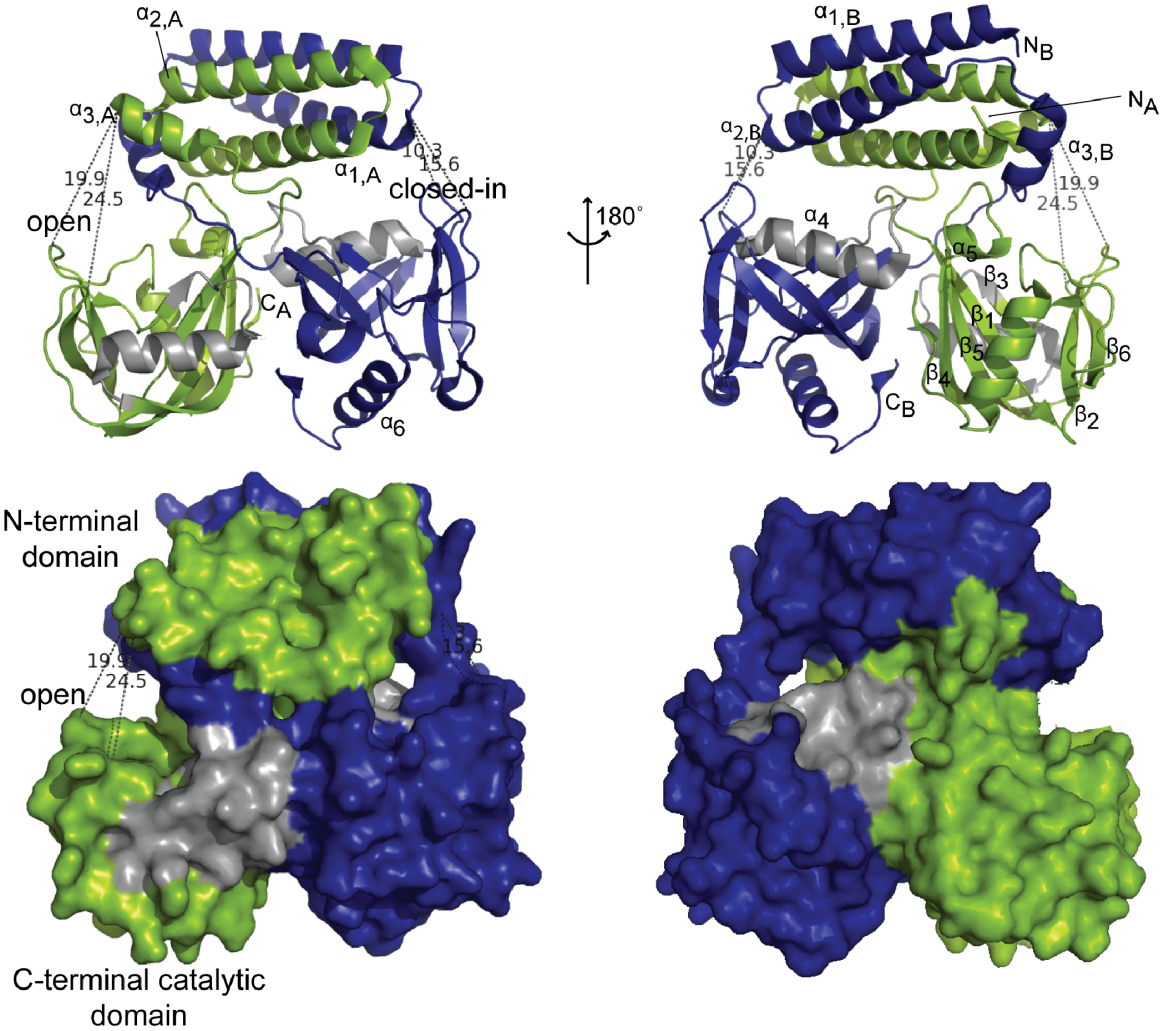
Structure of UDP-X diphosphatase. Top: Two alternate views of the structure of UDP-X diphosphatase. Monomer A is colored green and Monomer B blue. Secondary structural elements are labeled for each monomer in the N-terminal domain and for the C-terminal domain of Monomer A. The N and the C-termini of each monomer are labeled with an N or a C and a subscript indicating the monomer. Bottom: Surface rendering of UDP-Xase corresponding to views shown in the top panel. Distances are shown (in Å) for the following pairs of residues: A Asp51 and A Gly89 (19.9); A Asp51 and A Glu162 (24.5); B Asp26 and B Gly89 (15.6); B Asp26 and B Glu162 (10.3).

The structure of the wild-type *S. pneumoniae* UDP-Xase, determined to 1.39 Å resolution (Table 2 and Figure 3; PDB ID 4HFQ), shows that the enzyme is a homodimer in agreement with size-exclusion chromatography (data not shown) and solution scattering data (see below). Formation of the dimer (Figure 3) buries a total surface area of 6230 Å^2^.

**Table 2:** Data collection and refinement statistics for UDP-X diphosphatase.

Parameter	Result for	
	UDP-X diphosphatase-native	UDP-X diphosphatase- HgCl_2_
** Space group**	P3_1_	P3_1_
** Number of mol. asu**	2	2
** Unit cell**		
**a×b×c (Å)**	78.0**×**78.0**×**64.7	77.6**×**77.6**×**65.0
**α, β, γ (°)**	α = β = 90, γ = 120	α = β = 90, γ = 120
** Resolution range (Å)** a	50.00–1.39 (1.44–1.39)	50.00–1.95 (2.02–1.95)
** No. of observed reflections**	469,804	182,809
** No. of unique reflections**	88,640	31,951
**Redundancy**	5.3 (3.9)	5.7 (5.2)
**Completeness (%)**	100.0 (100.0)	100.0 (100.0)
**R_sym_^b^**	5.7 (49.6)	12.2 (55.4)
**Mean ** ***I/σI***	27.6 (2.4)	16.7 (2.6)
** Refinement statistics**		
** Resolution range (Å)**	50.00–1.39	
** Number of reflections**	84,083	
**Cutoff**	F >0σF	
**R**	0.170	
**R_free_ (5% of data)**	0.196	
** No. of atoms refined**		
** Protein**	3,529	
** Water**	532	
** Other**	173	
**Ramachandran plot**		
** % in allowed regions**	95.4	
** % in additional favored regions**	4.6	
** RMSD from ideal geometry**		
** Bond lengths (Å)**	0.010	
** Bond angles (°)**	1.37	
** Mean B factors (Å^2^)**	16.4	
** Protein**	13.6	
** Water**	29.9	
** Other**	28.9	
** Wilson B-factor (Å^2^)**	12.5	

a Data in parentheses correspond to the outermost resolution shell.

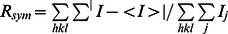
where <I> is the mean intensity of observations from a reflection and its symmetry equivalents.

Each monomer, monomer A (green in Figure 3) and monomer B (blue in Figure 3), consists of two domains: an N-terminal domain (residues 1–59) and a C-terminal catalytic domain (residues 69–203) connected by a loop (residues 60–68). The C-terminal catalytic domain contains the Nudix signature sequence that encodes residues involved in Mg^2+^ binding and catalysis [4]. This motif forms a loop-α-helix-loop structure (grey in Figure 3). The overall structure of the C-terminal catalytic domain is similar to that of other Nudix hydrolases but is most similar to that of human NUDT18 (PDB ID 3GG6; unpublished). Other Nudix hydrolases that share this fold include *B. cereus* CDP-Chase [5], *Bdellovibrio bacteriovorax* BdRppH [23], *E. coli* DHNTPase [38], and *E. coli* GDPMK [39].

The two C-terminal catalytic domains form a symmetric dimer stabilized by an extensive network of salt bridges and hydrogen bonds formed by Gln200, Leu127, Asp203, Thr107, Phe146, Gly103, Tyr104, Asp132, and Gln142. Different classes of quaternary arrangements have been observed for dimeric Nudix hydrolases, as exemplified by the arrangements of *E. coli* ADPRase [40] and *E. coli* GDPMH [41]. The quaternary structure of the C-terminal catalytic domain dimer in UDP-Xase more closely resembles that of *E. coli* GDPMH [41], [42].

The N-terminal domain of each monomer consists of 3 helices (α_1_–α_3_) separated by loops. The helix-loop-helix (HLH) motif consisting of the α_1_ and α_2_ helices and the intervening loop of each monomer form a “U”-shaped structure. The two HLH motifs stack against each other to form a four-helix bundle (Figure 3). The approximate two-fold axis of symmetry relating the N-terminal HLH motifs does not coincide with the two-fold axis of the C-terminal catalytic domain dimer, making UDP-Xase an asymmetric homodimer. The four-helix bundle is stabilized through interactions of residues facing the center of the helical bundle (Leu36, Met13, Met43, and Tyr33 of both monomers as well as Phe6 of monomer B) and through interactions involving residues of adjacent helices in the four-helix bundle (Tyr9 and Glu42 of both monomers and Arg12 and Gln46 of monomer B). In addition there is a network of hydrogen bonding interactions at the ends of the bundle. At the “U”-shaped turn of the HLH motif, Tyr33, Lys25, Thr24, and Asp29 of both monomers as well as Arg32 and Asp26 of monomer B are involved in hydrogen bonding interactions. Residues involved in hydrogen bonding interactions at the other end include Met 1 of both monomers, Lys2, Thr3, and Asp5 of monomer A and Gln46, Ser48, Asp49, Asp51, and Glu54 of monomer B. In the asymmetric homodimer, both catalytic sites–defined by the location of the Nudix motif– are located at the interface between the N- and C-terminal domains. This interface is stabilized by an extensive network of salt bridges and solvent-mediated hydrogen bonding networks involving Glu57, Thr62, Ala66, and Val102 of both monomers, Arg12, Leu138, Gln139, Ser140, Lys141, Tyr143, and Lys180 of monomer A and Asp35, Val58, Lys60, Pro61, Ser63, Ala64, Tyr65, Glu101, and Tyr104 of monomer B. As a consequence of the asymmetry at this interface, the two active sites of the homodimer are different: one site is “closed-in” forming a channel like structure (Figure 3), the other site is more “open” and exposed (Figure 3).

### RNase Activity of UDP-X diphosphatase

**Figure 4:**
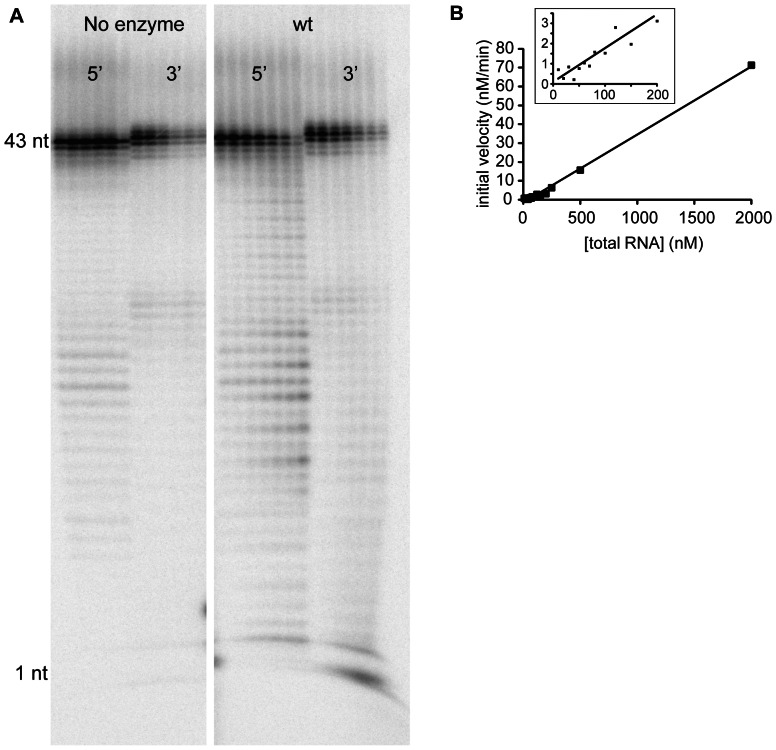
RNA exonuclease activity of UDP-X diphosphatase. (A) Assay of RNA exonuclease activity. RNA was labeled with [^32^P]-monophosphate on either the 5′-end or the 3′-end. Activity was measured in the absence (lanes 1–6 for 5′-end labeled RNA and lanes 7–12 for 3′-end labeled RNA) or presence (lanes 13–19 for 5′-end labeled RNA and lanes 20–26 for 3′-end labeled RNA) of UDP-Xase. In the absence of UDP-Xase, the six lanes represent timepoints of 0, 5, 10, 20, 30, and 60 min, from left to right. In the presence of UDP-Xase, the seven lanes represent timepoints of 1, 2, 5, 10, 20, 30, and 60 min (left to right). (B) Initial rates from competition assay with 20 nM 5′-labeled RNA substrate and varying amounts of unlabeled substrate. Inset: Zoomed-in view of the 0–200 nM RNA region of the plot.

Given its similarity to other RNA binding and processing proteins, we carried out experiments to determine whether the *S. pneumoniae* UDP-Xase degrades RNA. Using either a 5′- or a 3′-radiolabeled RNA as the substrate in an exonuclease assay, we analyzed the degradation products by denaturing gel electrophoresis (Figure 4A). The 5′-labeled RNA produced a laddering of degradation products. In contrast, the 3′-labeled substrate showed only accumulation of the 3′-labeled product at the bottom of the gel (Figure 4A). These results indicate that UDP-Xase degrades RNA in the 3′ 5′ direction. This activity is similar to that of the *B. cereus* CDP-Chase [5].

Competition assays in which the degradation of a radiolabeled RNA was measured in the presence of unlabeled RNA (Figure 4B) allowed measurements of RNA exonuclease activity at total RNA concentrations that were much higher than what is possible to measure using only radiolabeled RNA. Rates for RNA concentrations up to 2000 nM total RNA (20 nM labeled RNA and 1980 nM unlabeled RNA) were measured by this procedure. This, however, was not sufficient to determine true K_M_ and V_max_ since the enzyme activity was not saturated, even at 2000 nM. However, the results indicate the K_M_ is at least 2000 nM, V_max_ is at least 11.8×10^−3^ U/mg, and k_cat_ is at least 0.286 min^−1^.

### UDP-Xase Active Sites for Nudix Substrate and for RNA Exonuclease Activity

**Figure 5:**
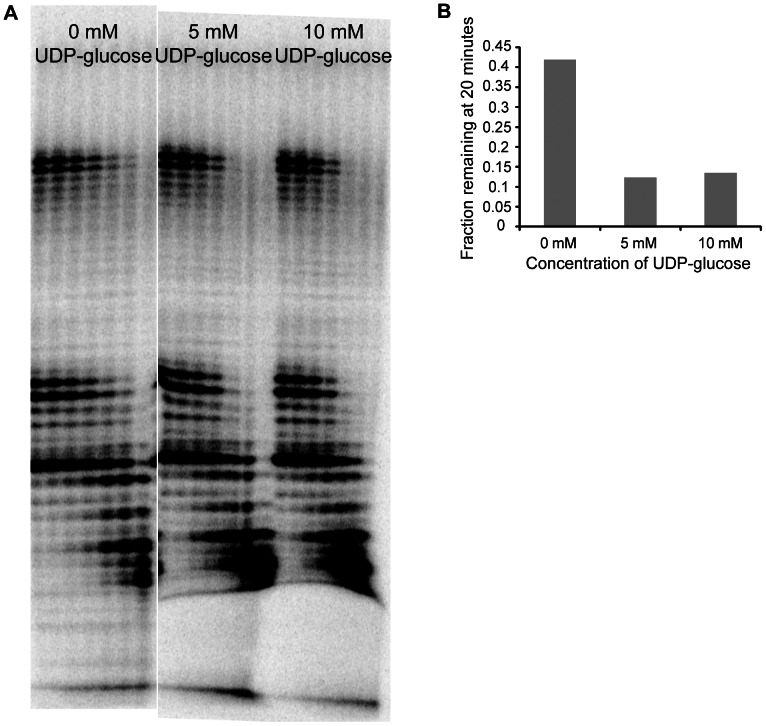
RNA exonuclease activity in the presence of UDP-glucose. (A) RNA exonuclease assay performed using 5′-[^32^P] labeled RNA with no UDP-glucose (lanes 1–7), 5 mM UDP-glucose (lanes 8–14), and 10 mM UDP-glucose (lanes 15–21). Lanes correspond to timepoints of 1 min, 2 min, 5 min, 10 min, 20 min, 30 min, and 60 min from left to right. (B) The fraction of 43 nt RNA remaining after 20 minutes at each UDP-glucose concentration is plotted.

The asymmetric dimer has two different Nudix active sites, each with the conserved Nudix box sequence. The “closed-in” site can accommodate a Nudix substrate but is too small to engage RNA. However, although the “open” site could potentially bind Nudix substrates, its cavity is large enough to accommodate an RNA molecule. To determine whether the Nudix activity and the RNA degradation activity use the same or different active sites, we performed a competition assay. Surprisingly, in the presence of saturating concentrations of UDP-glucose, the RNA exonuclease activity of UDP-Xase is accelerated (Figure 5). This suggests that not only do the two substrates use different active sites but also that the binding of a Nudix substrate at one active site produces a conformational change that increases RNA exonuclease activity at the other site. This observation could be the result of a conformational change in UDP-Xase that occurs upon binding of a Nudix substrate that optimizes the interactions between an RNA substrate and UDP-Xase at the other site. Given the distinct structural characteristics of the two sites, we propose that RNA binds at the “open” site and UDP-glucose and other Nudix substrates bind at the “closed-in” site.

**Figure 6:**
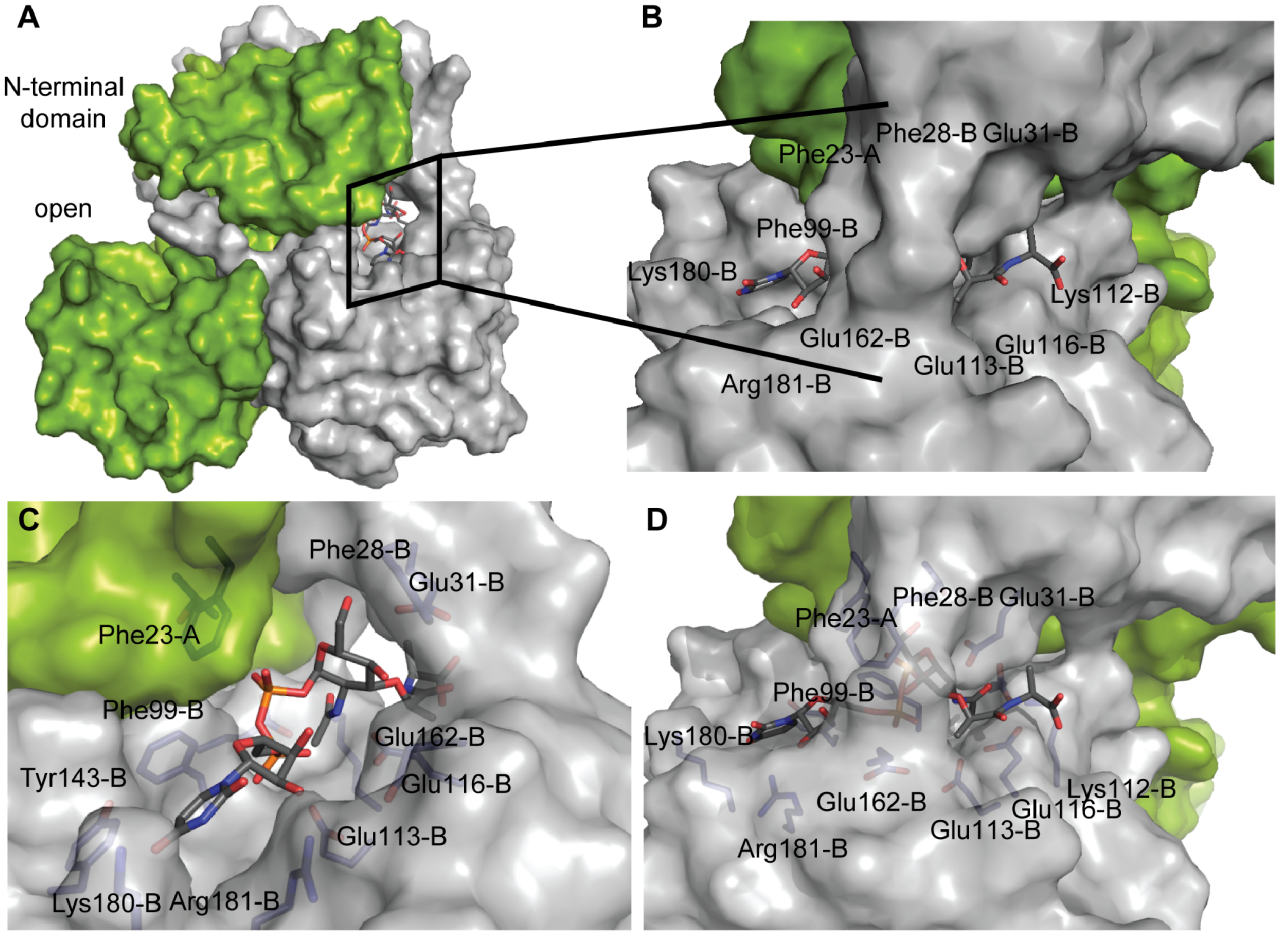
Model of the complex of UDP-Xase and UDP-N-acetylmuramoyl-L-alanine binding. (A) Monomer A is shown in green and monomer B is shown in grey. UDP-N-acetylmuramoyl-L-Ala is shown in sticks where grey represents carbon atoms, blue nitrogen, red oxygen, and orange phosphorus. (B) Close-up view of UDP-N-acetylmuramoyl-L-alanine binding channel. Residues lining the channel are labeled. (C) Close-up view, rotated 90° from B, of UDP-N-acetylmuramoyl-L-alanine binding channel with transparent surface. (D) Same as in B, but with transparent surface highlighting residues that line the UDP-N-acetylmuramoyl-L-alanine binding channel.

To further examine whether Nudix substrates bind to the “closed-in” site, we manually docked UDP-N-acetylmuramoyl-L-alanine into the “closed-in” site (Figure 6). The conformations of the residues surrounding the substrate were optimized by energy minimization (see Materials and Methods). The “closed-in” site, formed by the N- terminal domain of monomer A and the C-terminal domain of monomer B of the UDP-Xase dimer, is ideally suited for binding UDP-N-acetylmuramoyl-L-alanine (Figure 6) and possibly longer Mur pathway substrates such as UDP-N-acetylmuramoyl-L-alanine-D-glutamate-L-lysine.

### E113A and E162A Mutations: Evidence for RNA Binding to the Open Site

**Figure 7:**
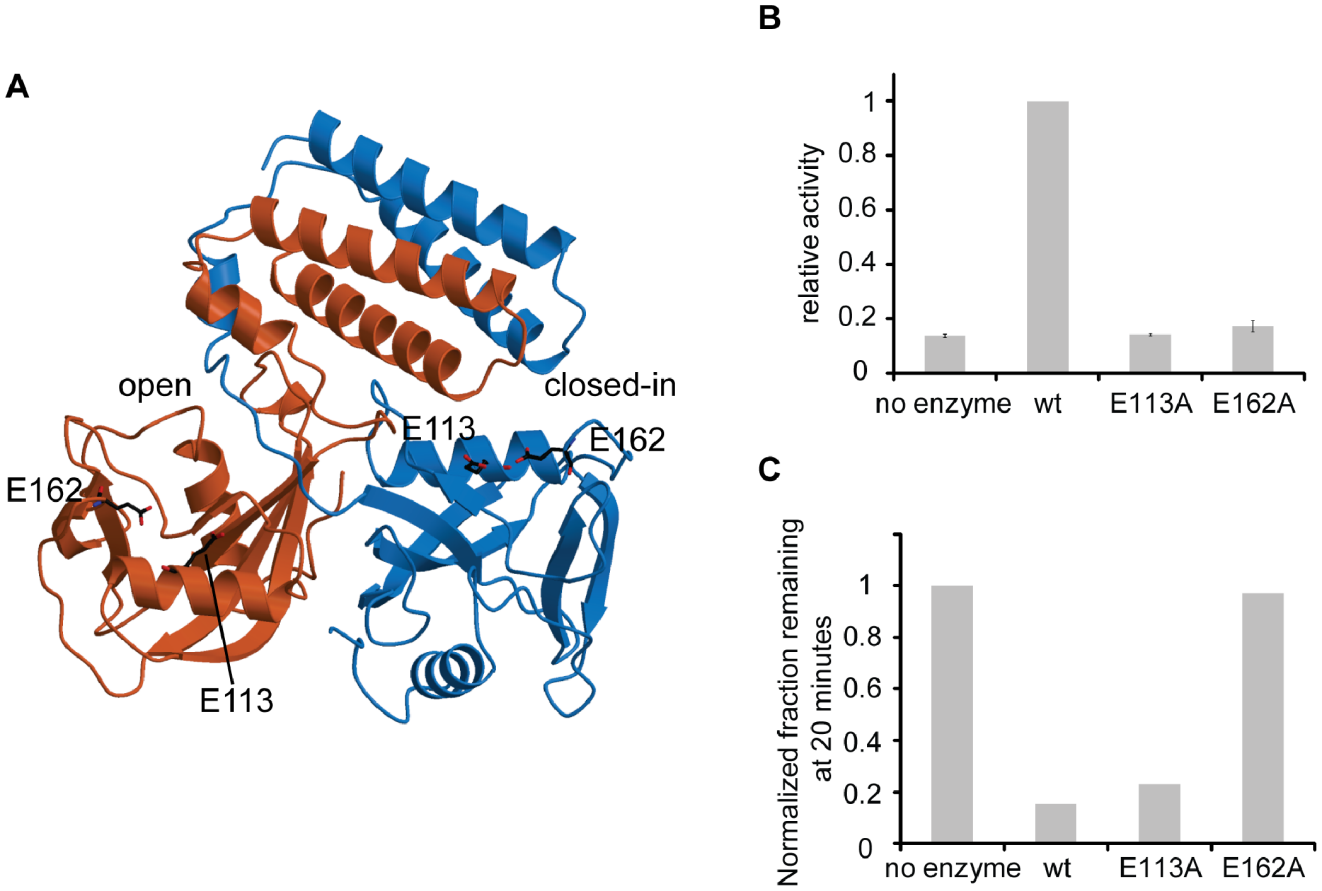
Effects of E113A and E162A mutations on UDP-glucose hydrolysis and RNA exonuclease activity. (A) Location of the E113A and E162A mutations. Monomer A is colored orange and monomer B blue. Residues are shown as sticks. (B) Activity of UDP-glucose hydrolysis. Activities were scaled to that of the wild-type enzyme. (C). RNA exonuclease activity. The fraction remaining 43 nt RNA after 20 minutes was normalized to the no enzyme control and plotted as a histogram. The gel is shown in Figure S2.

While we proposed that RNA binds to the cleft created by the “open” site, it is also possible that RNA could bind to another cleft of UDP-Xase that we have not identified. To further explore the possibility that the RNA exonuclease activity occurs at the “open” site, we mutated to alanine two conserved glutamate residues (Glu113 and Glu162) that typically participate in coordinating divalent metal cations required for Nudix catalytic activity (Figure 7A). Residue Glu113 of UDP-Xase corresponds to position 14 of the conserved residue of the Nudix motif and is typically involved in coordinating a catalytic metal in the Nudix active site. Glu162 is outside the Nudix box and was chosen because it is homologous to a residue that completes the coordination of a metal in the active site of other Nudix enzymes. As expected, mutation of either residue abolishes UDP-glucose hydrolysis, suggesting that Nudix catalytic activity requires both Glu113 and Glu162 (Figure 7B). Mutation of Glu 113 to alanine did slow down RNA exonuclease activity compared to the wild-type while mutation of Glu162 to alanine strongly diminished RNA exonuclease activity (Figure 7C and Figure S2). These results suggest that RNA indeed binds to the “open” site of UDP-Xase where the elements of the Nudix catalytic machinery may be used in a way that is different from their use in Nudix substrate hydrolysis.

### Small Angle X-ray Scattering (SAXS) of the E113A and E162A Mutants

**Figure 8:**
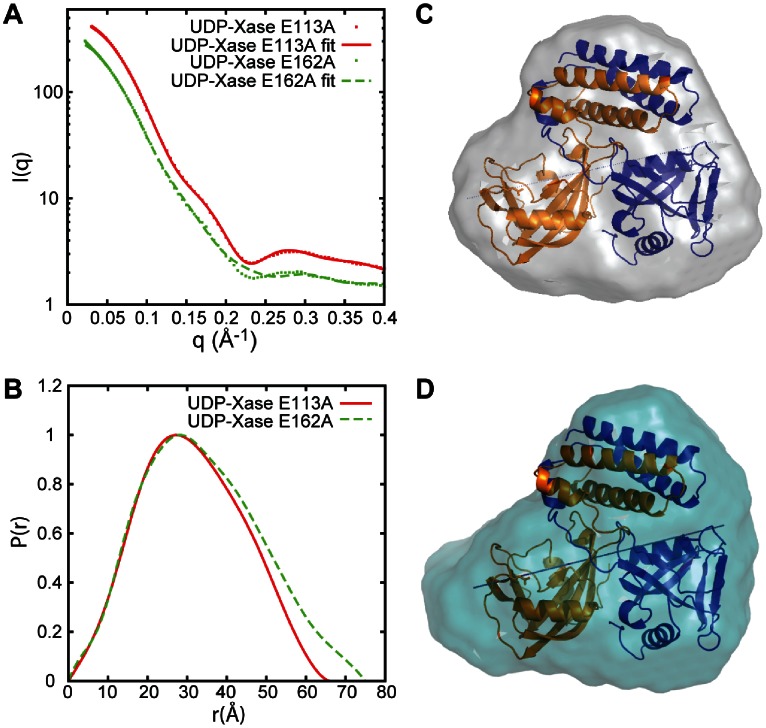
SAXS analysis of the UDP-Xase mutants. (A) Scattering data of the UDP-Xase E113A and E162A. (B) P(r) function derived from the SAXS profile in of UDP-Xase E113A and E162A. (C) Averaged *ab initio* envelope calculated by DAMMIN of the UDP-Xase E113A mutant superimposed with the wild-type UDP-Xase structure. (D) Averaged *ab initio* envelope of the UDP-Xase E162A mutant superimposed with th wild-type UDP-Xase structure.

As shown above, the Glu162 residue plays a critical role in both activities of UDP-Xase whereas the Glu113 residue is critical only to the Nudix catalytic activity. To understand their different effects on activity, we analyzed the E113A and E162A mutants using SAXS (Figure 8). Glu162 is in the loop that connects strands β5 and β6 (residues 153–162). In the E162A mutant, residue 162 does not coordinate the divalent cation and the loop may move to a position farther away from the rest of the structure, as observed in other Nudix enzymes [5]. The R_g_ and D_max_ calculated from the wild-type UDP-Xase crystal structure are 23.9 Å and 65.6 Å, respectively; the R_g_ and D_max_ calculated from the SAXS data of the mutants show that E113A has a conformation similar to that observed in the crystal structure of the wild-type (Table 3; R_g_ = 23.9 Å, D_max_ = 65.0 Å). In contrast, the E162A mutation results in an increase in R_g_ and D_max_ (26.1 Å and 75.0 Å, respectively; Table 3), suggesting that this mutation produces a conformational change.

**Table 3:** Structural parameters calculated from SAXS and goodness of fit obtained.

Sample	R_g_ (Å)	D_max_ (Å)	χ^2^
UDP-Xase E113A	23.9	65.0	2.26
UDP-Xase E162A	26.1	75.0	5.70

To better understand these conformations, we calculated molecular envelopes of UDP-Xase and fitted them with the crystal structure of UDP-Xase (PDB ID 4HFQ). The goodness of fit (χ^2^) of the models to the experimental SAXS data is given in Table 3. The molecular envelope of the E113A mutation computed from the SAXS data is perfectly suited to contain the dimer observed in the crystal structure of UDP-Xase, indicating that the dimer observed in the crystal and the dimer of the E113A mutant present in solution in the SAXS experiment are highly similar. The molecular envelope of the E162A mutation, however, has a slight protrusion not explained by the crystallographic dimer. In the wild-type UDP-Xase, the side chain of Glu162 points toward the Nudix catalytic site and participates in coordinating the metal cofactors. The protrusion in the molecular envelope of the E162A mutant is consistent with conformational flexibilitiy of the loop between strands β5 and β6, which contains the E162A mutation; taken together with the increased R_g_ and D_max_ for the E162A mutation, this envelope indicates that this loop adopts a conformation in which the side chain of E162 points away from the Nudix catalytic site. This possibility is supported by the fact that the structures of other Nudix enzymes also display conformational flexibility in this loop.

### Identification of a Family of Nudix Hydrolases that Function at the Cell Wall

The gene *SP_1669* of *S. pneumoniae* is located within an operon containing two genes that are crucial in cell wall biosynthesis and is upstream of an operon known to be involved in cell division. Both processes are closely linked: invagination during cell division has been found to be coordinated with the synthesis of peptidoglycan for cell wall biosynthesis [35]. Interestingly, UDP-Xase, the enzyme encoded by *SP_1669*, hydrolyzes the Mur pathway substrates UDP-N-acetylmuramic acid and UDP-N-acetylmuramoyl-L-alanine.

A structurally similar Nudix enzyme, CDP-Chase, hydrolyzes CDP-choline and is expressed during sporulation at the periphery of the developing spore where it may regulate phosphocholine concentrations [5]. Phosphocholine is incorporated into the cell wall as lipoteichoic and teichoic acid moieties in Gram positive bacteria [43]. Like UDP-Xase, CDP-Chase has also RNA exonuclease activity.

**Figure 9:**
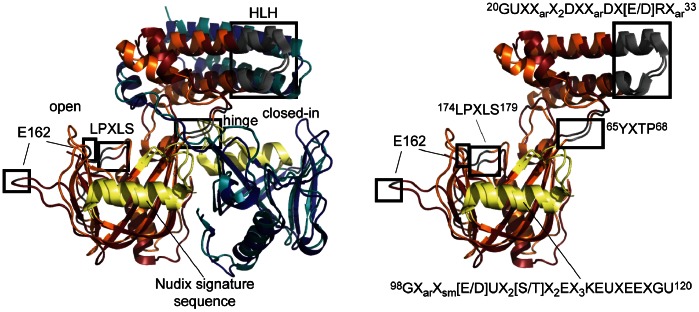
Structural superposition of the *S.* *pneumoniae* UDP-Xase and *B. cereus* CDP-Chase. (A) UDP-Xase in orange (monomer A) and blue (monomer B); CDP-Chase in redbrick (monomer A) and teal (monomer B). The conserved HLH motif of the N-terminal domain, the hinge between the N-terminal and Nudix domain, E162, and the LPXLS motif are shown in grey ribbons with black boxes. The Nudix signature sequence is shown in pale yellow ribbons. (B) Superpositon of only Monomer A of *S. pneumoniae* UDP-Xase and *B. cereus* CDP-Chase in the same orientation as in A. Conserved sequence motifs corresponding to boxes in A and the Nudix signature sequence are shown explicitly. Sequence numbering corresponds to that of UDP-Xase (see Figure 10).

**Figure 10:**
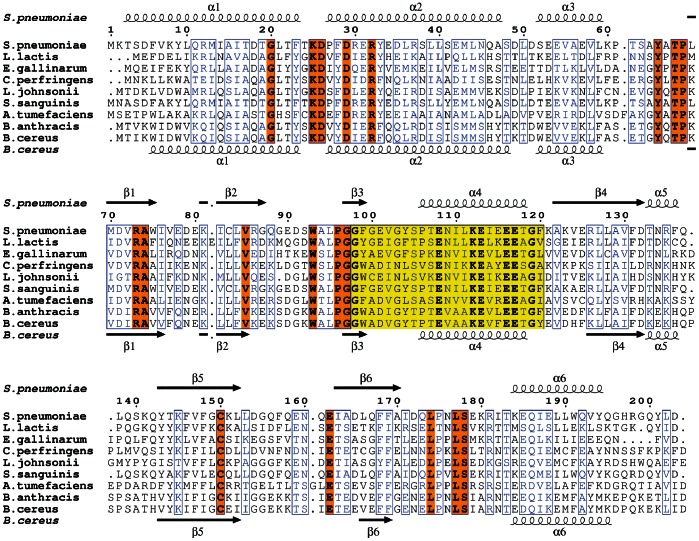
Alignment of putative Nudix hydrolases with high sequence homology to UDP-Xase and CDP-Chase. The yellow box indicates residues of the Nudix signature sequence. Absolutely conserved residues are in bold. Conserved residues characteristic of this subfamily that are outside the Nudix signature sequence are highlighted in orange. Similar residues are outlined in blue.

*S. pneumoniae* UDP-Xase and *B. cereus* CDP-Chase have highly similar folds (Figure 9; RMSD of 2.00 Å over 360 Cα carbons). Most residues outside the Nudix box that are conserved between these two proteins participate in stabilization of the four-helix bundle of the N-terminal domain, the Nudix fold, or the interface between the N- and C-terminal domains (Figure 9). These same conserved residues outside the Nudix motif are found in several putative Nudix hydrolases (Figure 10) that are present only in Gram positive bacteria. These enzymes are 200–210 amino acids in length and contain the motifs GUXX_ar_X_2_DXX_ar_DX[E/D]RX_ar_ in the HLH region (positions 20–33 in the sequence of UDP-Xase; U is a hydrophobic amino acid I, L, V; X is any amino acid, and X_ar_ is an aromatic amino acid), YXTP in the hinge/loop region between the N-terminal domain and the C-terminal domain (positions 65–68), and LPXLS (positions 174–179) in addition to a Nudix signature sequence in the form of GX_ar_X_sm_[E/D]UX_2_[S/T]X_2_EX_3_KEUXEEXGU (positions 98–120; X_sm_ is a non-bulky amino acid, generally A or G) (Figures 9 and 10).

We propose that these enzymes fold as asymmetric homodimers that contain two distinct active sites, an “open” active site which carries out a 3′→5′ exonuclease activity and a “closed-in” active site that carries out Nudix substrate hydrolysis. The formation of this asymmetric dimer is a hallmark feature of these Nudix hydrolases. Formation of an asymmetric homodimer is rare but is often biologically significant [44]. In this particular Nudix hydrolase family, elements of each monomer of the asymmetric dimer contribute to each of the two distinct active sites. A similar phenomenon has been observed for the NSP3 protein where two monomeric proteins fold to form an asymmetric homodimer, resulting in a single substrate binding site [45].

We also propose that members of this novel Nudix hydrolase family function close to the cell wall and are involved in processes such as peptidoglycan biosynthesis, cell division, and sporulation. All of these biological processes are linked in bacteria. The Nudix enzymatic activity and the RNA exonuclease activity may be present in a single enzyme as a way of coordinating activities that are needed simultaneously at a crucial stage of the cell cycle.

## Supporting Information

Figure S1 **SDS-PAGE of UDP-Xase.** SDS-PAGE analysis of wt, E113A, and E162A UDP-Xase. 2–5 µg of each protein was loaded on a Novex 4–12% Bis-Tris gel (Invitrogen).
(TIF)

Figure S2 **Effects of E113A and E162A mutations on RNA exonuclease activity.** RNA exonuclease assay reaction products visualized on a urea denaturing gel: 5′-[^32^P] labeled RNA with no enzyme (lanes 1–6), UDP-Xase wild-type (lanes 7–13), UDP-Xase E113A (lanes 14–20), and UDP-Xase E162A (lanes 21–27). Lanes correspond to the same time points as in Figure 4A.
(TIF)
