# Supplementary figures and images for: A UDP-X Diphosphatase from Streptococcus pneumoniae Hydrolyzes Precursors of Peptidoglycan Biosynthesis
Source: PLoS One. 2013 May 15;8(5):e64241. doi: 10.1371/journal.pone.0064241 (PMC3655063; doi:10.1371/journal.pone.0064241)

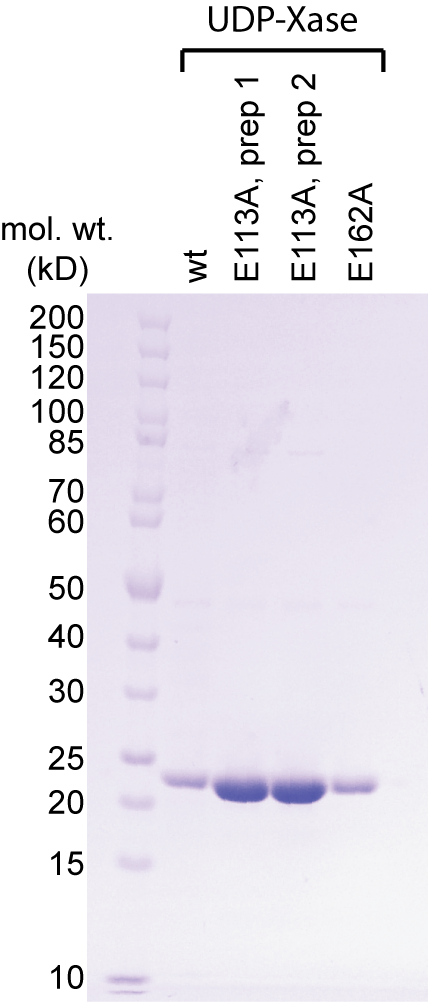

Supplement: Figure S1 — SDS-PAGE of UDP-Xase. SDS-PAGE analysis of wt, E113A, and E162A UDP-Xase. 2–5 µg of each protein was loaded on a Novex 4–12% Bis-Tris gel (Invitrogen). (TIF) [file pone.0064241.s001.tif]

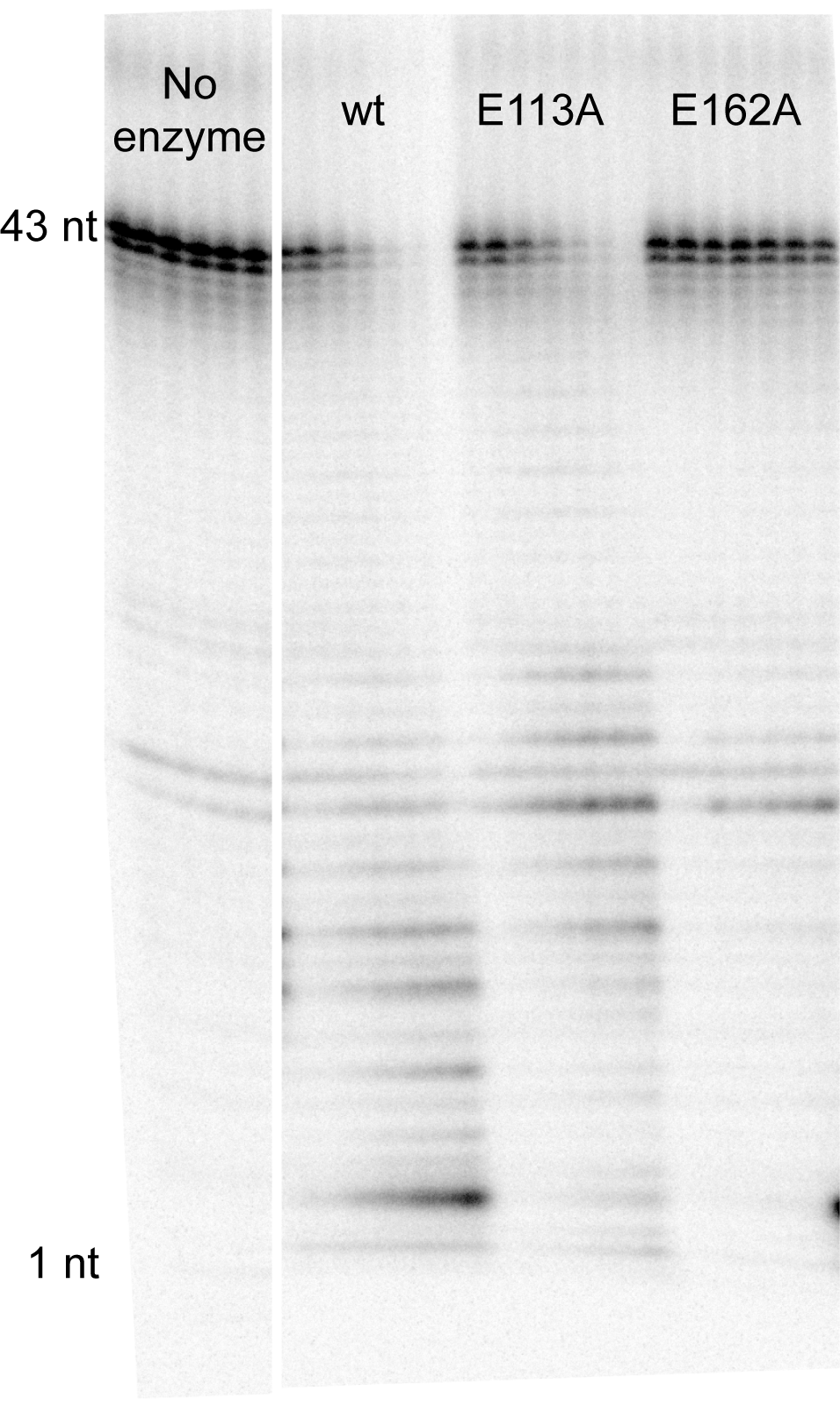

Supplement: Figure S2 — Effects of E113A and E162A mutations on RNA exonuclease activity. RNA exonuclease assay reaction products visualized on a urea denaturing gel: 5′-[32P] labeled RNA with no enzyme (lanes 1–6), UDP-Xase wild-type (lanes 7–13), UDP-Xase E113A (lanes 14–20), and UDP-Xase E162A (lanes 21–27). Lanes correspond to the same time points as in Figure 4A. (TIF) [file pone.0064241.s002.tif]
